# A Randomized Evaluation of Prophylactic Phenylephrine and Left Uterine Displacement for the Reduction of Hypotension After Spinal Anesthesia in Cesarean Delivery

**DOI:** 10.7759/cureus.102074

**Published:** 2026-01-22

**Authors:** Nighat Begum, Soma Butt, Gouhar Munir, Wazir Zeeshan Haider, Muhammad Ishaq, Shabbar H Changazi, Muhammad Imran

**Affiliations:** 1 Anaesthesiology, RHQ (Regional Headquarters) Teaching Hospital Gilgit, Gilgit, PAK; 2 Anaesthesiology, Farooq Hospital, Lahore, PAK; 3 Anaesthesiology, Services Hospital, Lahore, PAK; 4 Anaesthesiology, Provincial Headquarter Teaching Hospital Gilgit, Gilgit, PAK; 5 General Surgery, Provincial Headquarter Teaching Hospital Gilgit, Gilgit, PAK; 6 General Surgery, Shaheed Saif ur Rehman Government Teaching Hospital, Gilgit, PAK

**Keywords:** cesarean section, hypotension, patient positioning, phenylephrine, spinal anesthesia, vasoconstrictor agents

## Abstract

Background

Spinal anesthesia-induced hypotension is a frequent and critical problem during cesarean section, carrying risks for both mother and fetus. Prophylactic vasopressors and mechanical manipulation of the uterus are common preventative methods. The primary aim of this study was to assess the effectiveness of prophylactic phenylephrine bolus versus 15° left lateral tilt in preventing maternal hypotension.

Material and methods

In this randomized comparative study, 144 women who were planned to undergo elective cesarean delivery under spinal anesthesia were divided into two groups. Group A (n=72) was given a prophylactic intravenous bolus of 100 mcg of phenylephrine immediately after spinal injection. In group B (n=72), patients were placed in a 15° left lateral tilt with an obstetric wedge. The only outcome was the occurrence of hypotension (systolic pressure 20% decrease from baseline).

Results

Hypotension occurred in 65.3% (n=47) of patients in the phenylephrine group and 54.2% (n=39) of patients in the left tilt group. The difference between these groups was not statistically significant (p=0.159). Patients with a BMI over 30 kg/m² showed a higher incidence of hypotension after spinal anesthesia. However, there was no statistically significant difference in hypotension rates between those receiving phenylephrine and those positioned in a 15° left lateral tilt within this subgroup. Similarly, low baseline systolic blood pressure (SBP; 100-120 mmHg) was also a significant predictor of hypotension in both groups.

Conclusion

There was no benefit of phenylephrine 100 mcg bolus over a 15° tilt in the prevention of spinal anesthesia-induced hypotension for elective cesarean delivery. Both methods are popular options, but they may not be enough as individual prophylactic measures, and this might indicate the necessity of combination or more aggressive therapy.

## Introduction

For elective cesarean delivery, the preferred anesthetic technique is spinal anesthesia due to its safety profile and effective surgical anesthesia, with the mother awake and not at risk for complications such as failed intubation or pulmonary aspiration [1]. The sympathetic block by spinal anesthesia, despite its benefits, almost invariably results in vasodilatation and a profound reduction in systemic vascular resistance. The consequence of this can be maternal hypotension, a common side effect occurring in up to 80% among parturients [2].

The effects of maternal hypotension are clinically significant. For the fetus, this may result in decreased uteroplacental perfusion and, therefore, fetal acidosis, which could lead to low Apgar scores [3]. For the mother, hypotension manifests as several undesirable symptoms, including nausea, vomiting, and dizziness. These symptoms are not only uncomfortable but can also disrupt the birthing process, potentially complicating both the anesthetic management and the overall experience of delivery [4]. The pathophysiology includes both arterial vasodilatation and venous pooling (with aortic compression by the gravid uterus in supine posture), with subsequently impaired venous return and cardiac output [5].

Various approaches have been employed to address maternal hypotension following spinal anesthesia for cesarean delivery. One of the foundational strategies is preoperative crystalloid loading, which involves administering intravenous fluids before the procedure. While this method is widely used, evidence suggests that fluid preloading alone is often insufficient to prevent significant drops in blood pressure [6]. In addition to fluid management, the prophylactic use of vasopressors has become increasingly common. Agents such as phenylephrine and ephedrine are administered to maintain vascular tone and support blood pressure. Among these, phenylephrine, a pure α1-adrenergic agonist, has emerged as the preferred vasopressor [7]. Mechanical interventions also play a critical role in the prevention of hypotension. Left uterine displacement, typically achieved by tilting the patient approximately 15 degrees to the left, is a non-pharmacological technique recommended to counteract aortocaval compression. By relieving this pressure, venous return increases, resulting in stabilization of maternal blood pressure [8].

Despite frequent use of both pharmacological prophylaxis with vasopressors and mechanical prophylaxis via a left lateral tilt, there remains considerable uncertainty in the literature regarding which approach yields superior outcomes. Some studies have favored vasopressors, while others have highlighted the effectiveness of mechanical positioning, contributing to the ongoing debate and variability in clinical practice [9,10]. Given the critical importance of selecting an effective initial strategy to optimize both maternal and fetal outcomes during cesarean delivery and to provide clear guidance for anesthetic management, this randomized comparative study was designed to directly compare the efficacy of a prophylactic phenylephrine bolus with that of the left lateral tilt. The aim was to determine which method is more effective in preventing hypotension following spinal anesthesia in women undergoing elective cesarean section.

## Materials and methods

This randomized comparative study was conducted at Provincial Headquarter Teaching Hospital, Gilgit, from March 2022 to September 2025 after obtaining ethical approval. A total of 144 American Society of Anesthesiologists (ASA) physical status II women, aged 15-40 years, scheduled for elective cesarean delivery under spinal anesthesia were enrolled. Written informed consent was obtained from all participants. For participants under 18 years of age, written consent was obtained from a parent or guardian in addition to the participant's assent. Exclusion criteria included pre-existing or pregnancy-induced hypertension, cardiovascular or cerebrovascular disease, known fetal abnormalities, multiple pregnancies, emergency cesarean sections, and any contraindication to spinal anesthesia.

The sample size was calculated based on an expected incidence of hypotension of 50% in the control (left tilt) group and a target to detect a 25% absolute reduction (to 25%) in the phenylephrine group. With an alpha error of 0.05 and a power of 80%, the required sample size was 64 per group. Accounting for a potential 10% dropout rate, a total of 144 participants (72 per group) were recruited. Using a computer-generated sequence and sealed envelope method, participants were randomly allocated into two equal groups (n=72 each). Group A (phenylephrine) received a prophylactic intravenous bolus of 100 mcg phenylephrine immediately after the intrathecal injection. Group B (left tilt) was positioned with a 15° left lateral tilt using a standardized obstetric wedge under the right hip prior to the administration of spinal anesthesia.

The patients were preloaded with 10 ml/kg Ringer's lactate in all cases. Routine anesthesia monitoring (non-invasive blood pressure, electrocardiogram, and pulse oximetry) was established. Baseline systolic arterial pressure (SAP) was calculated as the average of three consistent measurements. Spinal anesthesia was administered with the patient positioned in the right lateral posture. A total of 2 ml of 0.75% hyperbaric bupivacaine was used to achieve the required anesthetic effect for the procedure. After the block, patients were all repositioned to their designated trial position (supine for Group A and 15˚ left tilt for Group B). SAP was monitored every five minutes from spinal anesthesia administration to fetal delivery, allowing prompt detection and management of hypotension.

The primary outcome was the occurrence of hypotension, defined as SAP reduction ≥20% from baseline or a value <100 mm Hg. If hypotension occurred in any participant, a 100-mcg intravenous phenylephrine bolus was administered as rescue therapy to promptly restore blood pressure. The data were processed using the software SPSS version 27.0 (IBM Corp., Armonk, NY, US). Descriptive variables such as age and BMI were presented as means with standard deviation, while categorical variables, such as hypotension, were presented as frequency and percentages. Baseline data were compared via an independent samples t-test. The chi-square test was employed for comparison of the primary outcome between the groups. A p-value < 0.05 was accepted as statistically significant. Post-hoc stratification was performed for age categories, BMI, and baseline SBP categories.

## Results

A total of 144 subjects were enrolled in the study. There were no significant differences in demographic characteristics between the two groups. Mean age was 26.61 ± 4.82 years and 28.53 ± 5.59 years in the phenylephrine group and left tilt group, respectively (p = 0.078, independent samples t-test). Similarly, the mean BMI was 28.4 ± 4.2 kg/m² in the phenylephrine group and 29.0 ± 4.5 kg/m² in the left tilt group (p = 0.42, independent samples t-test).

The primary endpoint of hypotension is shown in Table 1. Hypotension was observed in 47 (65.3%) of patients in the phenylephrine group and 39 (54.2%) of patients in the left tilt group. However, the difference was not significant.

**Table 1:** Incidence of hypotension following spinal anesthesia P-value and χ² statistic are from the Pearson chi-square test.

Group	Hypotension Present, n (%)	Hypotension Absent, n (%)	Total	χ² value	P-value
Phenylephrine	47 (65.3%)	25 (34.7%)	72	1.98	0.159
Left Tilt	39 (54.2%)	33 (45.8%)	72	-	-
Total	86 (59.7%)	58 (40.3%)	144	-	-

Stratified analysis showed that the incidence of hypotension remained high across most subgroups, and there was no significant difference between the phenylephrine group and the left tilt group in any stratum. The incidence of hypotension was highest in patients with BMI >30 kg/m² (phenylephrine 79.2% and left tilt 72.2%); however, this difference was not significant (p=0.610). Likewise, the incidence of hypotension was greater than 80% in both groups for the patients with a baseline SBP <120 mmHg (SBP between 100 and 120 mmHg). Young, lean, or higher baseline SBP patients had lower and similar rates between the groups (Table 2).

**Table 2:** Incidence of hypotension stratified by age, BMI, and baseline SBP categories *χ²* and P-values compare the incidence of hypotension between the phenylephrine and left tilt groups within each category by the chi-square test.

Stratification Variable	Category	Hypotension Present, n (%) Phenylephrine	Hypotension Present, n (%) Left Tilt	Total Present, n (%)	χ² value	P-value
Age (years)	15–30 (N=98)	38/55 (69.1%)	26/43 (60.5%)	64/98 (65.3%)	0.84	0.361
	31–40 (N=46)	9/17 (52.9%)	13/29 (44.8%)	22/46 (47.8%)	0.29	0.592
BMI (kg/m²)	<20 (N=18)	4/8 (50.0%)	3/10 (30.0%)	7/18 (38.9%)	0.82	0.365
	20–30 (N=86)	24/40 (60.0%)	19/46 (41.3%)	43/86 (50.0%)	3.02	0.083
	>30 (N=40)	19/24 (79.2%)	12/16 (75.0%)	31/40 (77.5%)	0.26	0.610
Baseline SBP (mmHg)	100–120 (N=68)	30/37 (81.1%)	25/31 (80.6%)	55/68 (80.9%)	0.003	0.956
	121–140 (N=62)	15/29 (51.7%)	12/33 (36.4%)	27/62 (43.5%)	1.44	0.232
	>140 (N=14)	2/6 (33.3%)	2/8 (25.0%)	4/14 (28.6%)	0.13	0.714

## Discussion

This randomized comparative study assessed the efficacy of two widely used prophylactic interventions for preventing hypotension following spinal anesthesia in women undergoing elective cesarean delivery. The interventions included an intravenous bolus of 100 mcg phenylephrine and a 15° left lateral tilt. The results demonstrated that neither the administration of a single 100 mcg bolus of phenylephrine nor the use of a 15° left lateral tilt led to a statistically significant reduction in the incidence of hypotension among the study participants. These findings underscore a critical point: employing either intervention alone is not sufficient to fully prevent spinal anesthesia-induced hypotension.

A notably high rate of hypotension was observed in the phenylephrine group, with 66.7% of patients affected. This result indicates that administering a single prophylactic 100 mcg phenylephrine bolus is, on its own, insufficient to reliably prevent hypotension associated with spinal anesthesia in this population. This finding is likely due to the pharmacokinetics of a one-time bolus. A 100-μg bolus may not be adequate to oppose the sudden and profound sympathectomy, which decreases by about 10 minutes after maximum block [11]. Recent evidence strongly favors continuous phenylephrine infusions, as they have been shown to not only be more efficient than bolus doses at maintaining stable hemodynamics [12]. For this reason, an international consensus statement in 2017 recommends prophylactic infusions compared to reactive bolus-only treatment [1].

The high incidence of hypotension (52.8%) seen in the left tilt group emphasizes that using outframed mechanical displacement by a 15° left lateral alone is not often sufficient to prevent spinal anesthesia-induced hypotension. The left tilt is crucial in evoking aortocaval pressure gradient, yet less crucial to attenuation of severe arterial vasodilatation after block from the head end to pressor-resistant levels [5]. In addition, the effectiveness of a tilt may be contingent on obtaining an adequate angle; MRI studies have shown that a 30° tilt or greater may be required to achieve significant volume change in the vena cava, a degree of tilt often infeasible for intra-operative access [13]. The use of a 15° wedge may be less than ideal on our part, though it is standard practice.

The stratified analysis showed significant modulators of hypotension risk. Greater BMI (>30 kg/m²) was correlated with higher rates of hypotension, albeit numerically greater within both groups (phenylephrine > left tilt), but the difference between interventions was not significant. This trend suggests that obesity may increase susceptibility to hypotension regardless of prophylactic strategy, possibly due to enhanced aortocaval compression, altered hemodynamics, or variable drug distribution. Low baseline SBP (100-120 mmHg) was also a significant predictor of hypotension, which may be explained by the fact that decreased baseline blood pressure is associated with decreased cardiovascular reserve [14]. These results suggest that patients with obesity or lower baseline BP may be less “protective” by either monotherapy and require more aggressive treatment or combined strategies to avoid hypotension.

Our findings are in line with a body of evidence indicating the importance of multimodal interventions. A meta-analysis by Heesen et al. showed that phenylephrine infusions are more effective than placebo for preventing hypotension during spinal anesthesia. The findings also suggest that combined prophylactic strategies are often needed, as using a single method alone is rarely sufficient [15]. The high rate of hypotensive events in each group in our study indicates that a protocol including crystalloid co-loading, prophylactic phenylephrine infusion, and simple left uterine displacement might be the best course rather than using just one modality [16].

This study has several important limitations. First, it was conducted at a single center, which may limit the generalizability of our findings. Second, the prophylactic intervention in the phenylephrine group was a single, fixed-dose bolus, which does not reflect the titrated, continuous infusion strategy currently recommended as best practice and may have contributed to the high rate of hypotension observed. Third, the 15° left lateral tilt, while standard, may represent a suboptimal degree of mechanical displacement; the use of a greater angle or manual uterine displacement was not assessed. Fourth, the absence of cardiac output monitoring precludes a deeper understanding of the underlying hemodynamic changes (e.g., changes in stroke volume or systemic vascular resistance) responsible for the hypotension, limiting mechanistic interpretation.

## Conclusions

In women undergoing elective cesarean delivery under spinal anesthesia, a prophylactic 100 mcg phenylephrine bolus was not superior to a 15° left lateral tilt in preventing hypotension. The high incidence of hypotension with both strategies indicates that neither is fully effective as a standalone prophylactic measure. Future protocols should combine a titrated phenylephrine infusion with mechanical displacement techniques to improve maternal hemodynamic stability and fetal outcomes.
